# Cannabis and creativity: highly potent cannabis impairs divergent thinking in regular cannabis users

**DOI:** 10.1007/s00213-014-3749-1

**Published:** 2014-10-07

**Authors:** Mikael A. Kowal, Arno Hazekamp, Lorenza S. Colzato, Henk van Steenbergen, Nic J. A. van der Wee, Jeffrey Durieux, Meriem Manai, Bernhard Hommel

**Affiliations:** 1 Cognitive Psychology Unit, Institute of Psychology, Leiden University, PO Box 9555, 2300 RB Leiden, The Netherlands; 2 Leiden Institute for Brain and Cognition, Leiden, The Netherlands; 3 Bedrocan BV, Veendam, The Netherlands; 4 Department of Psychiatry, Leiden University Medical Center, Leiden, The Netherlands

**Keywords:** Cannabis, Creativity, Divergent thinking, Convergent thinking

## Abstract

**Rationale:**

Cannabis users often claim that cannabis has the potential to enhance their creativity. Research suggests that aspects of creative performance might be improved when intoxicated with cannabis; however, the evidence is not conclusive.

**Objective:**

The aim of this study was to investigate the acute effects of cannabis on creativity.

**Methods:**

We examined the effects of administering a low (5.5 mg delta-9-tetrahydrocannabinol [THC]) or high (22 mg THC) dose of vaporized cannabis vs. placebo on creativity tasks tapping into divergent (Alternate Uses Task) and convergent (Remote Associates Task) thinking, in a population of regular cannabis users. The study used a randomized, double-blind, between-groups design.

**Results:**

Participants in the high-dose group (*n* = 18) displayed significantly worse performance on the divergent thinking task, compared to individuals in both the low-dose (*n* = 18) and placebo (*n* = 18) groups.

**Conclusions:**

The findings suggest that cannabis with low potency does not have any impact on creativity, while highly potent cannabis actually *impairs* divergent thinking.

## Introduction

Anecdotal evidence suggests that cannabis intoxication enhances human creativity. In line with that, Steve Jobs, an undeniably creative mind, once stated: “The best way I could describe the effect of the marijuana and hashish is that it would make me relaxed and creative.” Other regular users claim that cannabis induces a state in which they experience unusual and original thoughts (Tart 1970). In a more recent review, over 50 % of users reported heightened creativity during cannabis intoxication (Green et al. 2003). This widespread perception of cannabis as a creativity-enhancer makes it important to verify whether cannabis actually induces these supposed effects. Delta-9-tetrahydrocannabinol (THC), the main psychoactive compound present in the *Cannabis sativa* plant, has been found to reduce inhibitory control (McDonald et al. 2003) and stimulate striatal dopamine (DA) release (Bossong et al. 2009; Kuepper et al. 2013). These features of THC intoxication, in turn, are expected to play a role in particular aspects of creative thinking (Akbari Chermahini and Hommel 2010; Hommel 2012). On the other hand, THC has been linked to the emergence of psychotic symptoms both due to acute administration (D’Souza et al. 2004), as well as in the long-term (Kuepper et al. 2010). As a result, the possible beneficial effects of using cannabis, if any, might not outweigh the potential risks associated with its abuse.

The concept of creativity is not very well defined, and there is no agreement on one particular measure how to assess it. While some authors consider the concept to refer to the product of creative activities, others take it to reflect the personality of the product’s creator (for an overview, see Runco 2007). To circumvent these difficulties, we restricted our analyses to two well-established creative processes and the respective classical assessment methods: divergent and convergent thinking (Guilford 1967). Divergent thinking takes place when people try to find as many solutions to a loosely defined problem as possible—a process often referred to as “brainstorming.” It is often assessed by means of Guilford’s (1967) Alternate Uses Task (AUT), which requires individuals to generate as many as possible uses for a common household item (such as a pen or book) as they can think of (e.g., reading it, using it as a doorstop, etc.). In contrast, convergent thinking takes place when trying to find the one possible solution to a very well-defined problem. This process is often assessed by means of Mednick’s (1962) Remote Associates Task (RAT), in which people are presented with three supposedly unrelated concepts (e.g., “time,” “hair,” “stretch”) and are requested to identify the one concept that can be related to all three of them (“long”). Research indicates that performance in AUT and RAT is not (strongly) correlated (Akbari Chermahini and Hommel 2010; Akbari Chermahini et al. 2012). Moreover, there is evidence that the two types of creative thinking are differently related to subcortical DA levels: While divergent thinking performance relates to markers of DA levels in the form of an inverted U-shape, convergent thinking performance displays a linear, negative correlation with DA markers (Akbari Chermahini and Hommel 2010). In addition, this dissociation of human creativity seems to correspond to the dual pathway to creativity model (De Dreu et al. 2008; Nijstad et al. 2010) suggesting that creative performance emerges from the balance between cognitive flexibility and cognitive persistence—two dissociable cognitive control functions (De Dreu et al. 2012).

With regard to the neural effects of THC, the link between creative thinking and DA appears to be particularly interesting. Administration of THC has been shown to indirectly induce DA release in the striatum (Bossong et al. 2009; Kuepper et al. 2013), and there is evidence that its chronic application can lead to dopaminergic hypoactivity in the long-term, especially if the onset of cannabis use is at a young age (Hoffman et al. 2003; Urban et al. 2012; Bloomfield et al. 2014). As divergent thinking performance is expected to be optimal with medium subcortical DA levels (Akbari Chermahini and Hommel 2010), one may suspect that THC can have a beneficial effect on this creative process, particularly in individuals with low dopaminergic functioning. This assumption is further supported by the fact that the reduction in inhibitory control, as observed in response to stimulation by pure THC (McDonald et al. 2003) and cannabis (Ramaekers et al. 2006; Ramaekers et al. 2009), has been related to dopaminergic functioning as well (Mink 1996). Reduced inhibitory control can be considered to reflect a cognitive control state with weak top-down guidance. Such a state should affect convergent and divergent thinking differently (Hommel 2012). As pointed out by Bogacz (2007), human decision-making and the retrieval of possible alternatives can be considered a process that emerges from the interaction of top-down guidance and low-level competition between alternatives. If so, convergent thinking, with its many top-down constraints targeting one single solution, would seem to require a control state that provides strong top-down guidance and strong local competition. In contrast, divergent thinking, with its loosely defined problem and its many solutions, seems to require a control state that provides weak top-down guidance and only little local competition (Hommel 2012). To the degree that THC indeed induces a control state with weak top-down guidance and local competition, it might thus be expected to improve divergent thinking, interfere with convergent thinking, or both (Hommel 2012; Colzato et al. 2012).

Unfortunately, the available research on the link between cannabis and creativity allows only for partial verification of these expectations. With respect to divergent thinking, one study showed that subjects intoxicated with joints (cannabis cigarettes) containing a low dose of THC (3 mg in total) displayed significantly enhanced performance on two divergent production tasks, compared to a group that received a higher THC dose (6 mg in total; Weckowicz et al. 1975). Curran et al. (2002) showed that, as compared to placebo, oral THC (7.5 and 15 mg) dose-dependently improved verbal fluency—an important aspect of divergent thinking (Guilford 1967), at least as assessed by the AUT. Improved verbal fluency performance was also found in a naturalistic study that showed beneficial effect of smoked cannabis (10 % THC on average) on divergent thinking to be restricted to users low in trait creativity (i.e., individuals that obtained a low score on a self-assessment questionnaire about achievements in different creative domains; Schafer et al. 2012). In addition to fluency, cannabis administration (joints containing 19 mg of THC) has also been shown to increase the number of original responses on a test of associative processes, in comparison to placebo (Block et al. 1992). In contrast, Tinklenberg et al. (1978) did not observe any improvement in performance during the Torrance Tests of Creative Thinking (TTCT; Torrance 1966), which is often assumed to tap into divergent thinking, after oral consumption of THC (a biscuit containing 0.3 mg/kg body weight of THC). Another study found decreased TTCT scores for fluency, flexibility, and elaboration after smoking a cannabis joint (containing 10 mg of THC) in regular cannabis users but not in first-time users (Bourassa and Vaugeois 2001). In summary, the methodological differences between the various studies aside, many but not all findings suggest that THC may induce a cognitive control state with weak top-down guidance, thus efficiently decreasing the competition between cognitive representations and enhancing divergent thinking (Hommel 2012; Colzato et al. 2012).

For convergent thinking, the evidence is even more limited. Weckowicz et al. (1975) observed a trend toward less efficient convergent thinking task after smoking joints containing a low dose of THC (3 mg in total) or a higher dose (6 mg in total), in comparison to both a placebo and a pure control group. However, the same study also found impaired convergent thinking but only for the high-dose condition. The most recent investigation found potentially detrimental effects of smoking cannabis (10 % THC on average) on RAT performance in a group of cannabis users assumed to be high in trait creativity (Schafer et al. 2012). Although the naturalistic approach of this study makes it difficult to account for specific dose-related differences, the results of the research of both Schafer et al. (2012) and Weckowicz et al. (1975) suggest that THC can disrupt the process of searching and converging on a single solution to a problem.

A number of the observed inconsistencies between studies might be due to differences with respect to THC dosage and method of administration, which, in turn, affects the bioavailability and the onset of action of the compound (Hazekamp et al. 2006). Moreover, an individual’s history of cannabis use needs to be identified before cognitive changes in response to THC can be predicted. Administration of joints (containing up to 39 mg of THC) to regular cannabis users has been found to produce no accuracy impairments on a test battery assessing several cognitive functions (Hart et al. 2001) and, more specifically, on tasks related to episodic and working memory (Hart et al. 2010). Furthermore, after smoking a cannabis joint (containing 500 μg/kg body weight THC), chronic users did not display any behavioral deficiencies on tasks assessing tracking performance and divided attention (Ramaekers et al. 2009) or changes in an event-related potential (ERP) reflecting early attentional processes (Theunissen et al. 2012), compared to infrequent users. In addition, regular cannabis users were shown to display reduced sensitivity to the psychotomimetic effects of THC (administered as an intravenous dose up to 5 mg; D’Souza et al. 2008). In contrast, inhibitory control has been found to be similarly impaired among both occasional and chronic users when intoxicated with cannabis (Ramaekers et al. 2009).

Accordingly, since research points to reduced cannabinoid receptor type 1 (CB_1_) density in the brains of regular cannabis users (Hirvonen et al. 2012), one may suspect that the tolerance of chronic users to some of the detrimental effects of THC is, to some extent, related to their dopaminergic functioning. Specifically, due to the concentration of CB_1_ receptors at gamma-aminobutyric acid (GABA) and glutamate neurons, CB_1_ receptor downregulation can influence the activity of these neurotransmitters (Hoffman et al. 2003). Because DA neurons are frequently co-localized with GABAergic and glutamatergic terminals, the dopaminergic deficiencies observed in chronic cannabis users may be explained by lasting, maladaptive modulation of DA by GABA and glutamate (Fattore et al. 2010; Fernández-Ruiz et al. 2010). If so, keeping in mind the inverted U-shape relationship between subcortical DA levels and divergent thinking performance (Akbari Chermahini and Hommel 2010) and the effect of THC on striatal DA release (Bossong et al. 2009; Kuepper et al. 2013), it may be expected that individuals with a relatively low level of dopaminergic functioning, such as regular cannabis users, are more likely to demonstrate enhanced performance on a divergent thinking task, provided that the THC dose is not excessively high. In contrast, in a population without long-term dopaminergic imbalances, such as healthy drug-naïve individuals, even a reasonably low dose of THC could stimulate DA production to the level that it exceeds the threshold for optimal performance. In the case of convergent thinking performance, which is best with low subcortical DA levels (Akbari Chermahini and Hommel 2010), it may be predicted that it will deteriorate in response to THC, irrespective of the dose and cannabis use history of the individual.

In order to examine these possibilities, we investigated the effect of two different doses of vaporized cannabis (containing 5.5 or 22 mg THC; see “Study drugs” section) and placebo on convergent and divergent thinking in a sample of chronic cannabis users, using a between-groups design. On the basis of the assumption that a low dose of cannabis can remove potential impairments caused by regular use (Weckowicz et al. 1975; Kelleher et al. 2004), we expected that participants intoxicated with a low dose of cannabis should display higher scores on a divergent thinking task, compared to placebo. Conversely, we predicted impairment of performance in the high-dose condition, in contrast to the low-dose and placebo conditions. In the case of convergent thinking, we expected that both doses of cannabis should impair this process, compared to placebo. In addition, since divergent thinking performance has been found to be related to an individual’s mood (Zenasni and Lubart 2011), we assessed perceived mood as a possible modulating factor.

## Materials and methods

The current study was part of a larger study which involved additional tasks and measurements.

### Participants

Power analysis was performed to assess the approximate number of subjects required for detecting medium (*d* = 0.5) or large effect sizes (*d* = 0.8). Consequently, with an expected sample size of 60, three conditions, and a set alpha of 0.05, the power to detect main effects with a medium or large effect size for a between-group ANOVA is 0.679 and 0.979, respectively. Calculations were made using the analysis program fpower (Friendly 2014).

Fifty-nine healthy regular cannabis users (52 males and 7 females) participated in the study in exchange for a small financial compensation. Subjects were recruited through advertisements on the internet, on community bulletin boards, and in coffee shops (outlets in which Dutch law permits the sale of small quantities of cannabis to consumers) and by word of mouth. Detailed demographic and substance use information is presented in Table 1. Written informed consent was obtained from all participants, after a complete explanation of the nature of the study. The study was approved by the Medical Ethics Committee of the Leiden University Medical Center.

**Table 1:** Demographic and substance use data for each experimental group

	Placebo	5.5 mg THC	22 mg THC	Significance level
*N* (male/female)	18 (18:0)	18 (17:1)	18 (13:5)	*p* = 0.019
Age	21.1 (2.4)	21.1 (2.1)	22 (2.5)	n.s.
IQ test score	7.8 (2.6)	7.3 (2.7)	7.4 (2.3)	n.s.
Monthly cannabis use	42.8 (31.3)	51.3 (52.6)	39.3 (27.8)	n.s.
Years of cannabis exposure	6 (3.1)	4.8 (1.9)	6.2 (2.6)	n.s.
Monthly alcohol use	26.2 (17.8)	23.7 (19.8)	18.8 (13.5)	n.s.
Years of alcohol exposure	5.3 (2.6)	4.8 (2.5)	6.9 (2.7)	n.s.
Monthly nicotine use	214.4 (207.7)	121.3 (140)	156 (185.3)	n.s.
Years of nicotine exposure	4.6 (3.8)	3.5 (4.2)	4.3 (4)	n.s.

Standard deviations in parentheses

*n.s*. non-significant difference, *Age* reported in years, *IQ test score* measured by a shortened version of Raven’s Standard Progressive Matrices, *Monthly cannabis use* consumption of cannabis cigarettes (joints), *Monthly alcohol use* consumption of alcohol units, *Monthly nicotine use* consumption of cigarettes

The participants were randomly assigned to one out of three experimental conditions: placebo, 5.5 mg or 22 mg THC. The groups were comparable in terms of age, substance use characteristics, and IQ test score. All subjects were required to be regular users (use cannabis at least four times a week, for a minimum of 2 years) and to be native Dutch speakers. The exclusion criteria were as follows: (1) history or presence of an axis I psychiatric disorder (DSM-IV; assessed with the use of the Mini International Neuropsychiatric Interview (M.I.N.I.); Lecrubier et al. 1997), (2) clinically significant medical disease, (3) use of psychotropic medication, (4) current or previous regular use of other drugs except cannabis (regular use defined as having used a drug more than four times in a lifetime), and (5) abuse of alcohol (more than 14 units a week). Compliance with the inclusion and exclusion criteria was assessed by means of self-report. Additionally, subjects were asked to refrain from caffeine, chocolate, and alcohol 12 h before the experimental session and not to use nicotine 2 h before the study. It was also not allowed to use cannabis within 2 days before the experiment. Participants’ compliance with these criteria was evaluated by means of a personal interview and the use of a saliva drug test, which detected the recent use of cannabis, morphine, or cocaine (Oral-View™ Saliva Multi-Drug of Abuse Test; Alfa Scientific Designs, Inc., Poway, CA, U.S.A.).

From the initial sample of 59 subjects, two male participants withdrew from the study before completing the two creativity tasks—one stated personal issues, while the other did not provide any explanation. Another subject experienced anxiety before cannabis administration and had to abort the experiment. In the case of adverse events related to drug administration, one participant reported anxiety, combined with fatigue and nausea, which prevented him from completing the tasks. Moreover, one female subject was excluded from the analysis due to lack of compliance to task requirements (i.e., she refused completing the tasks due to not liking their nature). This left 54 subjects for the final analysis (48 males and 6 females), except for the convergent thinking task (RAT). In this case, one male participant (in the 22 mg THC condition) requested to abort the study due to personal reasons before being able to complete the task, which left only 53 data sets for the RAT analysis.

### Study drugs

The active drug substance consisted of the dried, milled, and homogenized flowers of the plant *C. sativa* (variety “Bedrocan”®; 19 % THC). It was obtained from Bedrocan BV (Veendam, The Netherlands) where it was cultivated under standardized conditions according to the requirements of Good Agricultural Practice (GAP). The placebo (variety “Bedrocan”®; <0.5 % THC) used in the study had a moisture content and terpenoid profile (providing the typical smell and taste of cannabis) identical to the active drug. Study medication was prepared by ACE Pharmaceuticals BV (Zeewolde, The Netherlands). For each individual dose, exact amounts of active cannabis and placebo were mixed so that each dose was equal to 250 mg total weight but with varying concentrations of THC (placebo/5.5 mg/22 mg THC). Study medication was stored in a refrigerator (2–8 °C) in triple-layer laminated foil pouches (Lamigrip). Shelf life stability under these conditions was determined to be at least 1 year.

On the study day, each subject received a randomized single dose of cannabis by means of a Volcano® vaporizer (Storz & Bickel GmbH, Tüttlingen, Germany)—a reliable and safe method of intrapulmonary administration of THC (Hazekamp et al. 2006; Zuurman et al. 2008). Cannabis was vaporized at a temperature of 230 °C into a standard Volcano balloon as supplied with the vaporizer. For blinding purposes, the Volcano balloon was covered with a non-transparent plastic bag so that no differences in density of the vapor were visible between dosages.

When administrating THC by means of vaporizing, it should be taken into account that only part of the dose present in the plant material is vaporized into the balloon (Hazekamp et al. 2006) and that a portion of the THC inhaled from the balloon is not absorbed by the lungs but is exhaled again (Zuurman et al. 2008). Therefore, in order to achieve an absorbed dose of approx. 2 and 8 mg THC, we loaded the Volcano vaporizer with 5.5 and 22 mg of THC, respectively. Moreover, since the THC delivery of the Volcano vaporizer and cannabis joints is comparable (Abrams et al. 2007), the loaded vs. absorbed dose distinction can be applied to smoked cannabis as well.

During administration, subjects were instructed to inhale deeply and hold their breath for 10 s after each inhalation. They were not allowed to speak during the inhalation period and were required to empty the balloon within 5 min. Subjects had the opportunity to practice the inhalation procedure using an empty balloon before cannabis administration.

### Shortened Raven’s Standard Progressive Matrices (measure of intelligence)

Individual IQ test scores were determined by means of a reasoning-based intelligence test (Raven et al. 1988). Each item of this test consists of a pattern or sequence of a diagrammatic puzzle with one piece missing, the task being to complete the pattern or sequence by choosing the correct missing piece from a list of options. The items are getting more difficult as the test taker proceeds through the test. The Standard Progressive Matrices (SPM) test assesses the individual’s ability to create perceptual relations and to reason by analogy independent of language and formal schooling. The version of the test used in the study consisted of 14 items.

### Alternate Uses Task (divergent thinking)

In this task (Guilford 1967), participants were asked to list as many possible uses for two common household items (i.e., pen, shoe) as they could. The scoring had four components: *fluency* (the total of all responses), *flexibility* (the number of different categories used; e.g., “household uses”), *originality* (where each response was compared to the responses from the other subjects, responses given by only 5 % of the participants being counted unusual [1 point] and responses given by only 1 % as unique [2 points]), and *elaboration* (referring to the amount of detail; e.g., while a book used as “a doorstop” would count 0, “a doorstop to prevent a door slamming shut in a strong wind” would count 2: 1 point for explanation of door slamming and 1 point for additional detail about the wind). Of these four criteria, the component *flexibility* has been found to be the theoretically most transparent and the empirically most consistent and reliable score (Akbari Chermahini and Hommel 2010).

### Remote Associates Task (convergent thinking)

In this task (developed by Mednick 1962), participants were presented with three unrelated words (e.g., time, hair, and stretch) and asked to find a common associate (long). The test consisted of 14 items, which were taken from Dutch version of the RAT from Akbari Chermahini et al. (2012).

### Affect grid (subjective measure of mood)

As in Colzato et al. (2013), the current mood of participants was assessed by means of a 9 × 9 Pleasure × Arousal grid (Russell et al. 1989).

### Visual analogue scales (subjective measure of drug effects)

The subjective effects of cannabis were assessed by means of three scales (horizontal 100-mm lines, the left pole labeled “not at all” and the right “extremely”) referring to “(feeling) high,” “good drug effect,” and “bad drug effect.” Participants were to mark a point at the continuous line to indicate their experience.

### Design and procedure

The study used a randomized, double-blind, placebo-controlled, between-groups (placebo vs. 5.5 vs. 22 mg THC) design. All participants were tested individually, and the order of the two creativity tasks—AUT and RAT—was counterbalanced. Upon arrival, the subjects were asked to complete the SPM test within 10 min. Afterward, the study drug was administered. Six minutes after cannabis administration, participants were required to indicate the subjective effects of the drugs by means of the visual analogue scales (VAS). This assessment of the effects of the drugs was then repeated twice—before and after the completion of the two creativity tasks (35 and 60 min after administration). Participants were provided with both the AUT and RAT in printed form (in the time window between 35 and 60 min after administration) and had 10 min to complete each task. In addition, in order to evaluate the subjective perception of mood, subjects were required to rate their mood on the Affect grid after the completion of each creativity task (at 48 and 60 min after administration).

### Statistical analysis

Scores from mood assessments and VAS, together with the five measures from the two creativity tasks (fluency, flexibility, originality, and elaboration scores from the AUT; the number of correct items from the RAT) were calculated for each subject. The results of the AUT were rated by two independent readers, blinded to the conditions (Cronbach’s alpha = 1.00 [fluency]; 0.87 [flexibility]; 0.94 [originality]; 0.9 [elaboration]). The final scores were the means of both ratings. All measures were analyzed separately. In the case of the AUT, RAT, and IQ test scores, age, and substance use data, between-groups ANOVAs were run with condition (placebo vs. 5.5 vs. 22 mg THC) as between-groups factor. Data regarding sex was analyzed with the use of a Pearson’s chi-squared test. Mood and VAS scores were analyzed by means of repeated-measures ANOVAs with time after cannabis administration (48 vs. 60 min. for mood; 6 vs. 35 vs. 60 min. for VAS) as a within-subjects factor and condition as a between-groups factor. Post hoc multiple comparisons *t* tests were applied with Bonferroni correction. A significance level of *p* < 0.05 was adopted for all tests.

## Results

### Demographic and substance use data

No significant main effects of condition were found in the case of age (*F*(2, 51) = 0.74, *p* = 0.482), IQ test score (*F*(2, 51) = 0.159, *p* = 0.854), monthly cannabis use (*F*(2, 51) = 0.453, *p* = 0.639), years of cannabis exposure (*F*(2, 51) = 1.433, *p* = 0.248), monthly alcohol use (*F*(2, 51) = 0.855, *p* = 0.431), years of alcohol exposure (*F*(2, 51) = 3.027, *p* = 0.057), monthly nicotine use (*F*(2, 51) = 1.231, *p* = 0.3), and years of nicotine exposure (*F*(2, 51) = 0.383, *p* = 0.684). However, the experimental conditions significantly differed by sex (*χ* ^*2*^(2, *N* = 54) = 7.875, *p* = 0.019); see Table 1.

### Creativity tasks

**Fig. 1:**
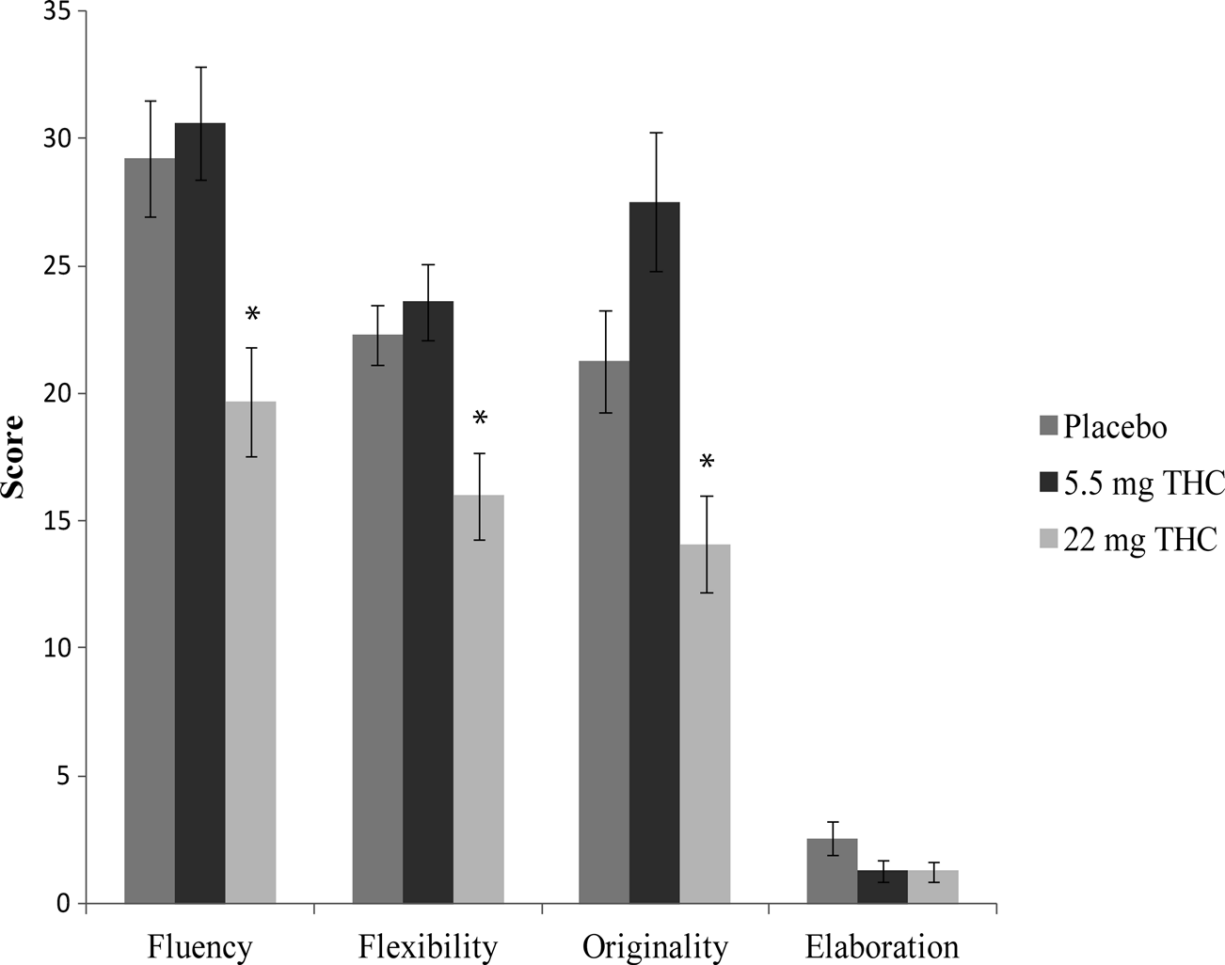
*Bar graphs* showing mean scores for the four components of the Alternate Uses Task (AUT; *fluency*, *flexibility*, *originality*, and *elaboration*) for each experimental group. The *asterisk* indicates a significant (*p* < 0.05) difference between the 5.5 and 22 mg THC conditions and between the placebo and 22 mg THC conditions. *Error bars* represent SE of the mean

Overall task performance in the AUT and RAT was comparable to studies without pharmacological interventions (e.g., Akbari Chermahini and Hommel 2010); see Fig. 1 and Table 2.

**Table 2:** Means, SD, and ANOVA results for the four components of the Alternate Uses Task (AUT; *fluency*, *flexibility*, *originality*, and *elaboration*) and the number of correct items from the Remote Associates Task (RAT), for each experimental group

	Placebo	5.5 mg THC	22 mg THC	*F* value	*p* value	*η* ^*2*^_*p*_	*MSE*
AUT							
Fluency*	29.2 (9.5)	30.6 (9.2)	19.6 (9)	7.378	0.002	0.224	86.615
Flexibility*	22.3 (4.9)	23.6 (6.2)	16 (7.2)	7.708	0.001	0.232	38.683
Originality*	21.2 (8.4)	27.5 (11.5)	14.1 (8.1)	8.952	<0.001	0.26	90.63
Elaboration	2.5 (2.8)	1.2 (1.6)	1.2 (1.6)	2.152	0.127	0.078	4.552
RAT	4.8 (2.3)	4.5 (2.8)	4.9 (3.6)	0.116	0.891	0.005	8.904

*p < 0.05 (significant difference between 5.5 and 22 mg THC, and between placebo and 22 mg THC)

#### Divergent thinking

Significant main effects of condition were found on fluency (*F*(2, 51) = 7.378, *p* = 0.002), flexibility (*F*(2, 51) = 7.708, *p* = 0.001), and originality (*F*(2, 51) = 8.952, *p* < 0.001), but not on elaboration (*p* > 0.05).

As expected, post hoc multiple comparisons revealed that participants in the 22 mg THC condition showed significantly reduced scores, as compared to placebo and 5.5 mg THC, respectively, for fluency (*t*(34) = 3.072, *p* = 0.01; *t*(34) = 3.582, *p* = 0.003), flexibility (*t*(34) = 3.061, *p* = 0.011; *t*(34) = 3.367, *p* = 0.002), and originality (*t*(34) = 2.584, *p* = 0.045; *t*(34) = 4.021, *p* < 0.001). However, contrary to expectations, subjects in the 5.5 mg THC condition did not display any significant increases over placebo, on any of the AUT components (*p* > 0.05).

Moreover, in order to test whether sex differences had impact on the observed results and match the groups for sex, we repeated the analysis after the exclusion of all female subjects. Significant main effects were retained for fluency (*F*(2, 45) = 5.774, *p* = 0.006), flexibility (*F*(2, 45) = 6.325, *p* = 0.004), and originality (*F*(2, 45) = 7.641, *p* = 0.001).

#### Convergent thinking

Contrary to expectations, there was no main effect of condition on the number of correct items from the RAT (*p* > 0.05).

### Subjective measures of drug effects and mood

#### Drug effects

Overall, only the rating of “high” showed a main effect of time after cannabis administration (with Huynh-Feldt correction; *F*(1.862, 93.109) = 15.777, *p* < 0.001). However, significant main effects of condition were found on all three scores: high (*F*(2, 50) = 11.656, *p* < 0.001), good drug effect (*F*(2, 50) = 8.701, *p* = 0.001), and bad drug effect (*F*(2, 50) = 6.507, *p* = 0.003). There were no significant interaction effects (*p* > 0.05).

**Fig. 2:**
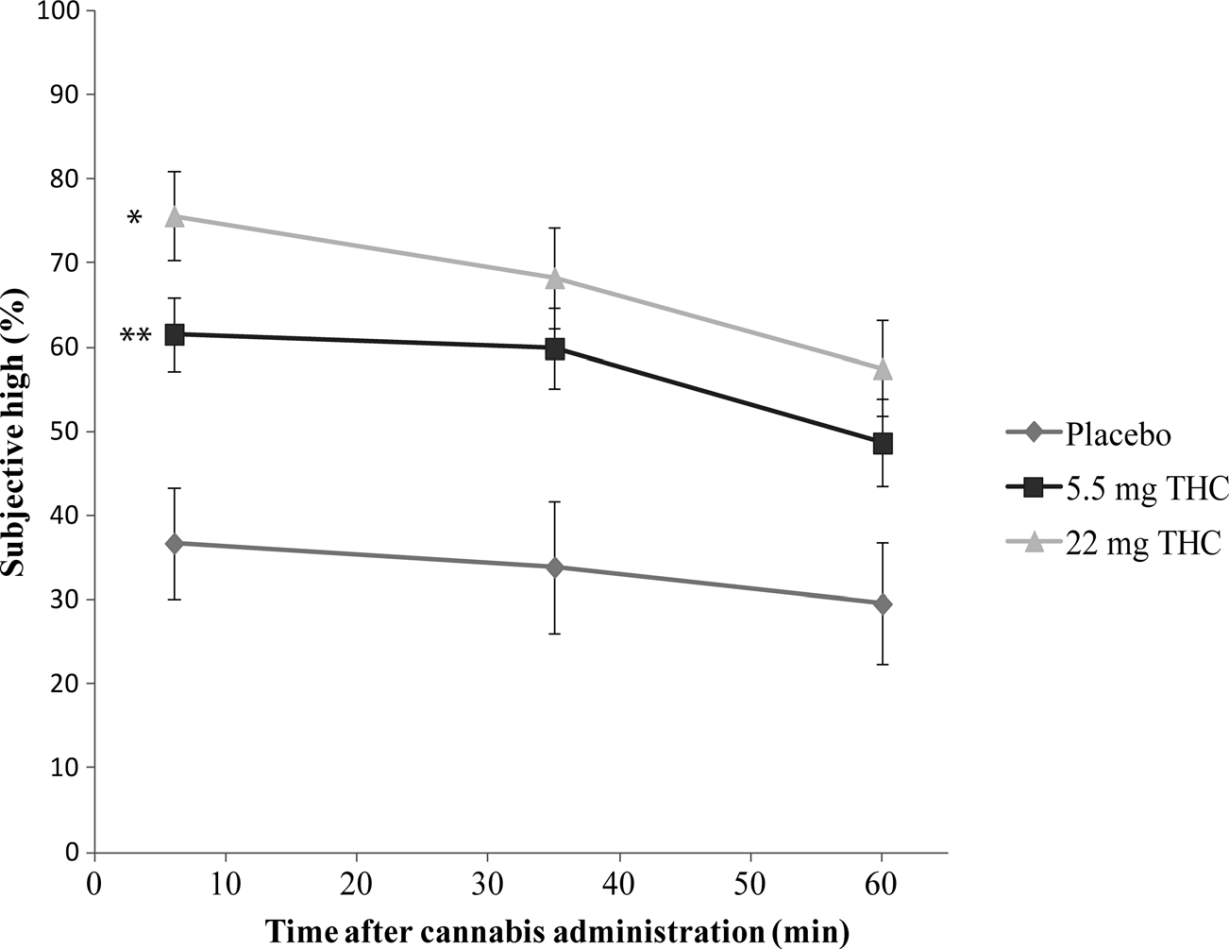
Mean subjective high (rated as a percentage) experienced in each experimental group as a function of time after cannabis administration. *Symbols* indicate a significant (*p* < 0.01) difference between the 22 mg THC and placebo conditions (*) and between the 5.5 mg THC and placebo conditions (**). *Error bars* represent SE of the mean

**Fig. 3:**
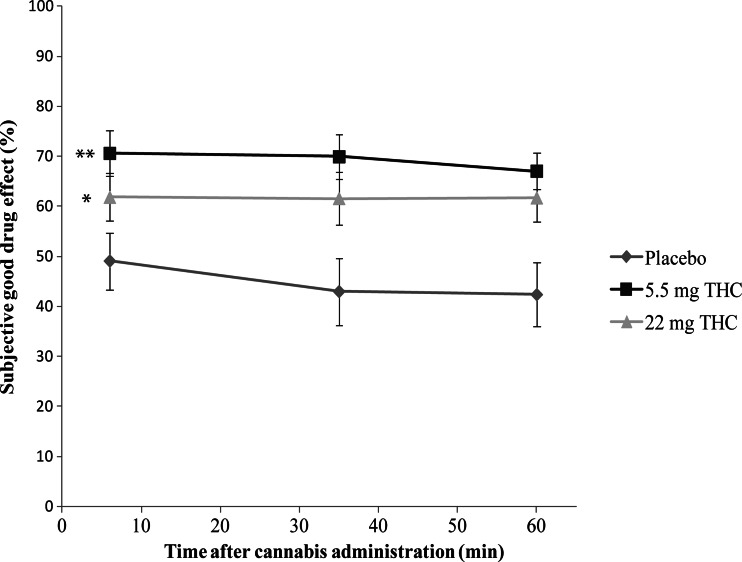
Mean subjective good drug effect (rated as a percentage) experienced in each experimental group as a function of time after cannabis administration. *Symbols* indicate a significant (*p* < 0.05) difference between the 22 mg THC and placebo conditions (*) and between the 5.5 mg THC and placebo conditions (**). *Error bars* represent SE of the mean

**Fig. 4:**
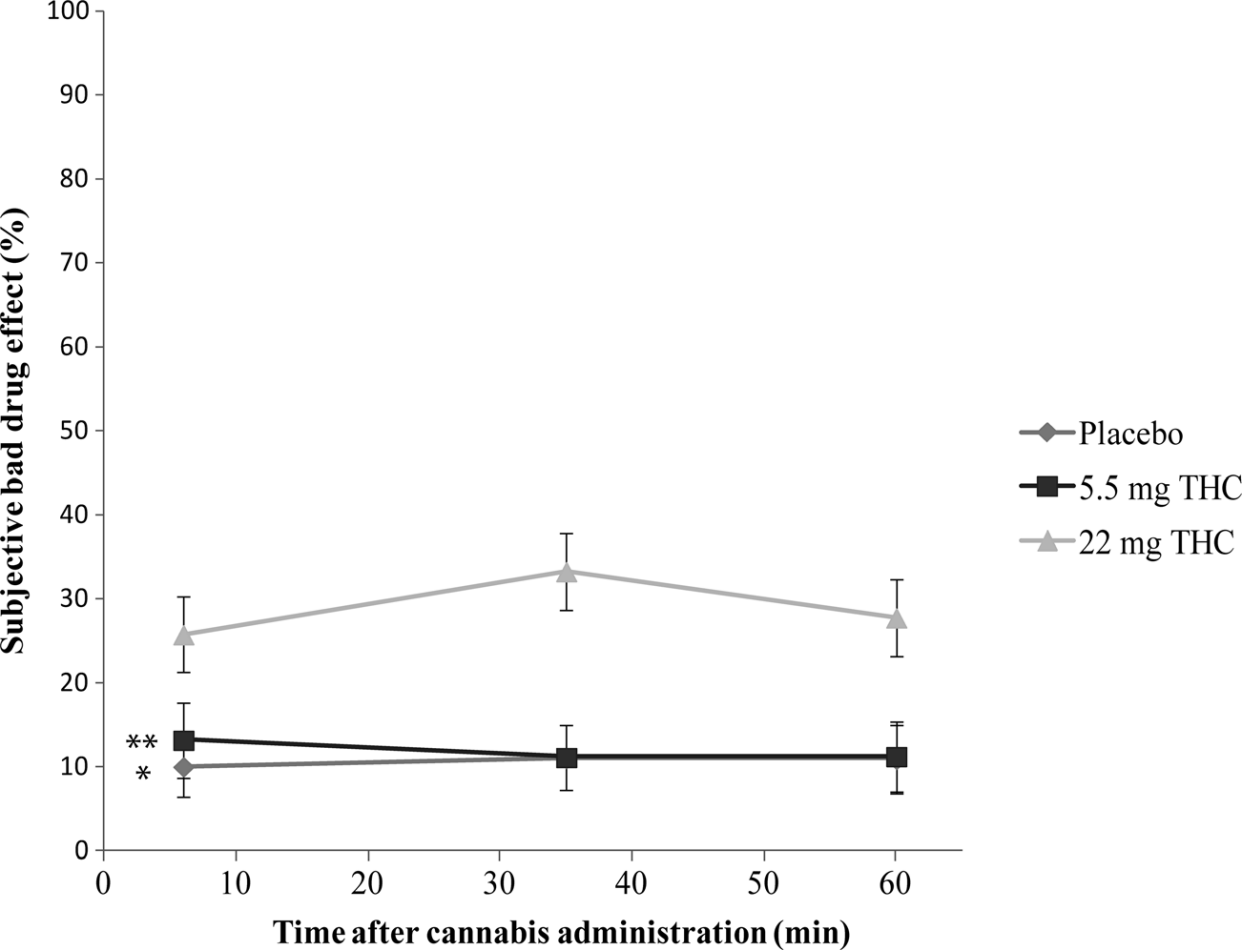
Mean subjective bad drug effect (rated as a percentage) experienced in each experimental group as a function of time after cannabis administration. *Symbols* indicate a significant (*p* < 0.05) difference between the placebo and 22 mg THC conditions (*) and between the 5.5 and 22 mg THC conditions (**). *Error bars* represent SE of the mean

Post hoc multiple comparisons revealed that subjects in the placebo condition showed significantly lower ratings of high, compared to the 5.5 mg (*t*(34) = 2.95, *p* = 0.006) and 22 mg THC groups (*t*(34) = 4.49, *p* < 0.001); see Fig. 2. Moreover, the ratings of good drug effect in the placebo condition were significantly lower than in the 5.5 mg (*t*(34) = 3.535, *p* < 0.001) and 22 mg THC groups (*t*(34) = 2.365, *p* = 0.023); see Fig. 3. In the case of both high and good drug effect, no significant differences were found between the scores in the 5.5 mg and 22 mg THC groups (*p* > 0.05). Conversely, in the case of the ratings of bad drug effect, participants in the 22 mg THC condition demonstrated significantly increased scores, compared to placebo (*t*(34) = 3.48, *p* = 0.006) and 5.5 mg THC (*t*(34) = 3.141, *p* = 0.012); see Fig. 4. In addition, the ratings of bad drug effect did not significantly differ between the placebo and 5.5 mg THC conditions (*p* > 0.05).

#### Mood

There were no main effects of time after cannabis administration on the ratings of pleasure or arousal (*p* > 0.05). Moreover, mood ratings in the placebo (6.3 vs. 6.2 for pleasure; 5.1 vs. 5 for arousal), 5.5 mg (7.1 vs. 7 for pleasure; 5.5 vs. 5.2 for arousal), and 22 mg THC (6.1 vs. 6.4 for pleasure; 4.8 vs. 4.7 for arousal) conditions did not show significant main effects of condition on pleasure or arousal (*p* > 0.05). There were no significant interaction effects (*p* > 0.05).

## Discussion

Our findings demonstrate that a high dose of vaporized cannabis (22 mg THC) impairs divergent thinking in regular cannabis users, in comparison to a low dose (5.5 mg THC) and placebo cannabis preparation. This is reflected in the decreased scores for fluency, flexibility, and originality of responses of participants in the high-dose condition. However, contrary to expectations, a low dose of cannabis did not enhance divergent thinking in chronic cannabis users: Individuals in the low-dose group did not significantly outperform subjects in the placebo group on any of the components of the AUT. Moreover, convergent thinking appears to be unaffected by either a low or high dose of cannabis, as condition had no impact on the numbers of correct RAT items.

Although the conclusions are limited by a between-groups design, the finding that administration of a high, but not low, dose of cannabis impairs divergent thinking performance of regular cannabis users may suggest that DA release in the striata of participants in the high-dose condition (Bossong et al. 2009; Kuepper et al. 2013) exceeded the threshold for optimal performance (Akbari Chermahini and Hommel 2010). This is in line with neuroscientific considerations that point to a homeostatic function of DA in regulating the balance between opposing cognitive control states—flexibility and stability (Cools et al. 2009; Cools and D’Esposito 2011). Flexibility refers to the ability to effectively switch between cognitive representations for the purpose of choosing the best alternatives, while the function of stability is to promote constancy of representations in spite of interference (Cools and D’Esposito 2011). Consequently, keeping in mind the effect of cannabis on inhibition (Ramaekers et al. 2006; Ramaekers et al. 2009), it is safe to assume that individuals in the high-dose condition experienced a reduction in inhibitory control after cannabis administration. Although this should promote a control state with weak top-down guidance allowing for flexible updating of information (Hommel 2012; Colzato et al. 2012), supra-optimal levels of DA in the striatum have been found to stimulate flexibility to the point that it surpasses the threshold for optimal performance, inducing distractibility as a result (see Cools and D’Esposito 2011). Accordingly, it is possible that the observed impairment of divergent thinking in the high-dose condition was the result of this process. Presumably, induction of a control state with weak top-down guidance is a necessary, but not sufficient, prerequisite for enhanced divergent thinking performance. Conversely, excessively potent cannabis may disturb the delicate balance between stability and flexibility by stimulating flexibility to its extreme, hence impairing divergent thinking.

In addition, from a more motivational perspective, it is possible that a high dose of cannabis induces the phenomenon of “ego-depletion” (i.e., exhausts the limited cognitive resources and motivation required for cognitive control operations; Baumeister et al. 1998; Inzlicht and Schmeichel 2012). This seems probable taking into account the observation that participants in the high-dose condition experienced more intense unpleasant subjective effects of cannabis, than those in the low-dose and placebo groups. In line with that, research points to anxiety, paranoia, delusions, and mental disorganization as frequent adverse effects of cannabis intoxication (Green et al. 2003; D’Souza et al. 2004). Therefore, the various undesirable forms of distraction induced by cannabis could have drained the control resources of individuals in the high-dose condition. In other words, it is possible that the need to exert self-control over the adverse effects of cannabis leads to a reduction in motivation and available cognitive resources required for subsequent optimal divergent thinking performance (Inzlicht and Schmeichel 2012).

In the case of the low-dose group, the lack of enhancement of divergent thinking does not provide support for the idea that a low dose of cannabis can eliminate cognitive impairments caused by regular use (Weckowicz et al. 1975; Kelleher et al. 2004). Nevertheless, since the performance of subjects in the low-dose and placebo groups was comparable in the case of the AUT, it may be assumed that the lack of cannabis-induced cognitive deterioration in the low-dose condition was indicative of the tolerance of regular cannabis users to the effects of the drug (Hart et al. 2001; Ramaekers et al. 2009; Hart et al. 2010; Theunissen et al. 2012). Furthermore, it is possible that the similar level of performance of both groups reflects their maximal potential for divergent thinking. Research indicates that placebo effects are able to stimulate subcortical DA release (Scott et al. 2007; Scott et al. 2008). Possibly, administration of a low dose of cannabis resulted in a comparable dopaminergic response as in the case of the placebo (Bossong et al. 2009; Kuepper et al. 2013). This seems plausible considering the fact that the placebo cannabis preparation used in the study was identical in terms of smell and taste to actual cannabis. As such, it had more potential to produce a placebo effect. In addition, the minimal amount of THC present in the placebo might have also affected DA release to some extent. Consequently, the subcortical DA levels of individuals in both the low-dose and placebo conditions could have been within the range for optimal divergent thinking performance (Akbari Chermahini and Hommel 2010).

### Limitations

Although the most recent investigation into the link between cannabis and convergent thinking suggested a potentially detrimental effect of cannabis intoxication on this process (Schafer et al. 2012), our study failed to detect any impact on RAT performance. Perhaps, our version of the task with 14 items was not sensitive enough to identify potential cannabis-induced impairments. Moreover, an important limitation is the between-groups design of the study. Consequently, it is possible that particular characteristics of the subject sample could have altered the effects of the drug. Specifically, the difference in sex between the conditions seems as a likely candidate in this regard (Crane et al. 2013). In addition, research points to genetic predispositions like polymorphism of the CB_1_ receptor gene (Beng-Choon et al. 2011; Stadelman et al. 2011), or the catechol-*O*-methyltransferase (COMT) gene (Schulz et al. 2012), as other factors which might modulate the cognitive effects of cannabis intoxication.

Another issue is related to the causal relation between the observed results and THC. In spite of the fact that application of cannabis, instead of pure THC, provides the benefit of a higher ecological validity of the study, the use of plant material could have influenced the findings. Specifically, terpenoids, which are the compounds responsible for the characteristic smell and taste of cannabis, have been shown to interact with cannabinoids to produce various synergistic effects (see Russo 2011). However, even if that was the case in our experiment, the terpenoid profile was comparable between the different doses, including the placebo cannabis preparation. Consequently, any potential terpenoid-cannabinoid interactions were controlled for. Unfortunately, the study lacked a measurement of THC blood plasma levels, which would allow for evaluating the relation between THC in the bloodstream and task performance. Furthermore, since the number of inhalations from the Volcano balloon and the duration of inhalations were not standardized, it is likely that this resulted in large differences in absorbed THC between subjects. In addition, the saliva test used in our experiment provided only an estimate of recent use. Possibly, the compliance of subjects with no-consumption criteria should instead be verified by examining the urinary levels of THC metabolites (11-COOH-THC), which is capable of detecting intoxication over a longer period of time. Moreover, the lack of testing for alcohol intoxication can be considered another limitation in evaluating the compliance of participants with no-consumption criteria.

### Conclusion

The findings indicate that administration of cannabis with a high THC content to regular cannabis users is detrimental for divergent thinking, while less potent cannabis does not seem to enhance this important component of creativity. The available evidence allows only for a speculation about the presence of these effects in a group of drug-naïve individuals, or occasional cannabis users. In any case, it can be claimed that the phenomenological experience of a person intoxicated with cannabis might not necessarily reflect his or her actual performance. In particular, the frequently reported feeling of heightened creativity could be an illusion. In other words, smoking a joint may not be the best choice when in need of breaking the “writer’s block,” or overcoming other artistic inhibitions, and smoking several of them might actually be counter-productive.
